# The use of clonidine in elderly patients with delirium; pharmacokinetics and hemodynamic responses

**DOI:** 10.1186/s40360-018-0218-1

**Published:** 2018-06-08

**Authors:** Karen Roksund Hov, Bjørn Erik Neerland, Anders Mikal Andersen, Øystein Undseth, Vegard Bruun Wyller, Alasdair M. J. MacLullich, Eva Skovlund, Eirik Qvigstad, Torgeir Bruun Wyller

**Affiliations:** 1 Oslo Delirium Research Group, Department of Geriatric Medicine, Oslo University Hospital, Oslo, Norway; 2 Institute of Clinical Medicine, University of Oslo, Oslo, Norway; 3 Department of Pharmacology, Oslo University Hospital, Oslo, Norway; 4 Department of Acute Medicine, Oslo University Hospital, Oslo, Norway; 5 Department of Paediatrics, Akershus University Hospital, Lørenskog, Norway; 6 Edinburgh Delirium Research Group, Geriatric Medicine, University of Edinburgh, Edinburgh, UK; 7 Department of Public Health and Nursing, Norwegian University of Science and Technology, Trondheim, Norway; 8 Department of Cardiology, Oslo University Hospital, Oslo, Norway

**Keywords:** Delirium, Clonidine, RCT, Pharmacokinetic

## Abstract

**Background:**

The Oslo Study of Clonidine in Elderly Patients with Delirium (LUCID) is an RCT investigating the effect of clonidine in medical patients > 65 years with delirium. To assess the dosage regimen and safety measures of this study protocol, we measured the plasma concentrations and hemodynamic effects of clonidine in the first 20 patients.

**Methods:**

Patients were randomised to clonidine (*n* = 10) or placebo (*n* = 10). The treatment group was given a loading dose (75μg every 3rd hour up to a maximum of 4 doses) to reach steady state, and further 75μg twice daily until delirium free for 2 days, discharge or a maximum of 7 days. Blood pressure (BP) and heart rate (HR) were measured just before every dose. If the systolic BP was < 100 mmHg or HR < 50 beats per minute the next dose was omitted. Plasma concentrations of clonidine were measured 3 h after each drug intake on day 1, just before intake (day 2 and at steady state day 4–6) and 3 h after intake at steady state (C_max_). Our estimated pre-specified plasma concentration target range was 0.3–0.7μg/L.

**Results:**

3 h after the first dose of 75μg clonidine, plasma concentration levels rose to median 0.35 (range 0.24–0.40)μg/L. Median trough concentration (C_0_) at day 2 was 0.70 (0.47–0.96)μg/L. At steady state, median C_0_ was 0.47 (0.36–0.76)μg/L, rising to C_max_ 0.74 (0.56–0.95)μg/L 3 h post dose. A significant haemodynamic change from baseline was only found at a few time-points during the loading doses within the clonidine group. There was however extensive individual BP and HR variation in both the clonidine and placebo groups, and when comparing the change scores (delta values) between the clonidine and the placebo groups, there were no significant differences.

**Conclusions:**

The plasma concentration of clonidine was at the higher end of the estimated therapeutic range. Hemodynamic changes during clonidine treatment were as expected, with trends towards lower blood pressure and heart rate in patients treated with clonidine, but with dose adjustments based on SBP this protocol appears safe.

**Trial registration:**

ClinicalTrials.gov NCT01956604, 09.25.2013. EudraCT Number: 2013–000815-26, 03.18.2013. Enrolment of first participant: 04.24.2014.

## Background

Despite their dominance in the clinical practice of medicine, older people are poorly represented in drug trials [1]. There are many potential reasons for this, including heterogeneity due to variations in biological aging, the wide range of comorbidities and polypharmacy. For informed decisions to be made in older people, however, it is important that drug trials overcome these challenges.

Delirium is a disturbance in attention, awareness and cognition with acute onset resulting from medical illness, trauma, surgery or drugs. Delirium affects 20% of hospitalised patients [2] and is associated with poor outcomes [3]. Drugs are widely used in the treatment of delirium [4, 5], despite the lack of positive evidence of their effectiveness [6, 7]. The pathogenesis is not well understood, but one prominent hypothesis is that delirium may in part result from exaggerated and/or prolonged stress responses [8].

Dexmedetomidine, a parenterally administered alpha2-adrenergic receptor agonist which attenuates sympathetic nervous system activity [9], shows promise as treatment for delirium in intensive care units (ICU) [10–16]. A recently published RCT showed that prophylactic low-dose dexmedetomidine significantly decreased occurrence of post-operative delirium [17]. Indeed, it is now in clinical use in the USA and Europe [18]. However, most patients with delirium are outside of ICUs, where intravenous use of dexmedetomidine is not feasible. An alternative agent is orally administered clonidine, which has very similar pharmacological properties to dexmedetomidine [19], even though its alpha-2-adrenergic selectivity is lower [20]. Clonidine in delirium is poorly studied, but a pilot study showed that the use of clonidine infusion during the weaning period after surgery for type-A aortic dissection might reduce the severity of delirium [21].

The Oslo Study of Clonidine in Elderly Patients with Delirium (LUCID) is an RCT designed to investigate the effectiveness of clonidine as a treatment for delirium in geriatric medical patients [22]. The properties of clonidine make it challenging to anticipate the right dosage to achieve an appropriate balance between efficacy and safety in older patients. Most pharmacological studies of clonidine have been conducted in younger adults [23–25] or in children [26, 27]. Concentrations of clonidine known to have clinical effects in adults range from 0.2 to 2.0 μg/L [24, 25, 27]. After oral administration the maximum plasma concentration (C_max_) occurs after 1–3 h [23]; higher doses yield proportionately higher concentrations [25]. The metabolism of clonidine is hepatic, mainly through CYP2D6 [28], but varying amounts of unmetabolised clonidine are excreted by the kidneys. The reduction in mean arterial pressure (MAP), as well as the risk of side effects like sedation and dry mouth, is highest when the plasma concentration of clonidine peaks (i.e. between 2 and 3 h after administration), even though only the hemodynamic effects (i.e. blood pressure and heart rate) correlate with the concentration levels [25].

To the best of our knowledge, there are no studies on the safety of clonidine and its hemodynamic effects in geriatric hospitalized patients with delirium. This study aims to investigate in detail the dosage regimen of clonidine in the LUCID protocol through measuring both plasma concentrations and hemodynamic effects. We aimed for the intermediate to low levels, that is, between 0.3 and 0.7 μg/L, because higher plasma concentration levels may increase the risk of adverse events, including hypotension, whereas plasma concentration levels lower than 0.3 μg/L might be insufficient to give a significant clinical effect.

## Methods

LUCID is a randomised, placebo-controlled, double-blind, parallel group study with 4-month prospective follow-up [22]. Acutely admitted medical patients > 65 years with delirium or subsyndromal delirium are eligible for inclusion. The selection criteria are presented in Table 1. Included patients were randomised to treatment with oral clonidine or placebo for a maximum of 7 days. The goal is to include 100 patients and the primary endpoint is the trajectory of delirium including measurements of attention, awareness and cognitive function. However, according to the pre-specified protocol, pharmacological analysis of clonidine and safety of the treatment will be assessed in the first 20 patients, before the final protocol for dosage regimen and safety assessments in the final 80 patients are decided. Our pre-specified target values were plasma-concentrations 0.3–0.7 μg/L, but a small number of measurements outside this range would be considered acceptable.

**Table 1:** Eligibility criteria

Inclusion criteria	
• Patient > 65 years old admitted to an acute medical ward • Delirium or subsyndromal delirium within the last 48 h • Signed informed consent from patient or relatives including advance consent for on-going treatment and follow up	
Exclusion criteria	
• Symptomatic bradycardia, bradycardia due to sick sinus syndrome, second- or third- degree AV-block (if not treated with pacemaker) or any other reason causing HR^a^ < 50 bpm^b^ at time of inclusion • Symptomatic hypotension or orthostatic hypotension, or a systolic BP^c^ < 120 at the time of inclusion • Ischemic stroke within the last 3 months or critical peripheral ischemia • Acute coronary syndrome, unstable or severe coronary heart disease (symptoms at minimal physical activity; NYHA^d^ 3 and 4) and moderate to severe heart failure (NYHA 3 and 4). (Acute coronary syndrome is defined according to international guidelines) • A diagnosis of polyneuropathy, phaeochromocytoma or renal insufficiency (estimated GFR^e^< 30 ml/min according to the MDRD^f^ formula) • Body weight < 45 kg • Considered as moribund on admission • Unable to take oral medications • Current use of tricyclic antidepressants, monoamine reuptake inhibitors or cyclosporine • Previously included in this study • Adverse reactions to clonidine or excipients (lactose, saccharose) • Not speaking or reading Norwegian • Any other condition as evaluated by the treating physician • Admitted to the intensive care unit	

^a^HR = heart rate

^b^bpm = beats per minute

^c^BP = blood pressure

^d^NYHA = New York Heart Association Functional Classification

^e^GFR = glomerular filtration rate

^f^MDRD = Modification of Diet in Renal Disease formula for estimated GFR

### Study medication

Each capsule (CAPSUGEL) contained either 75 μg Catapresan (clonidine hydrochloride) or 75 μg placebo, and was produced and labelled by “Kragerø tablettproduksjon A/S”.

### Dosage plan and safety

A loading dose (one capsule every 3rd hour up to a maximum of 4 doses on day 1) was given to achieve steady state. Further dosage was one capsule twice daily (8 am and 8 pm) until delirium free for 2 days, discharge or a maximum of 7 days treatment, whichever came first (see Table 2). Blood pressure (BP) and heart rate (HR) was measured just before every dose. The capsule was not given if the systolic BP (SBP) was < 100 mmHg or HR < 50 beats per minute (bpm). Serum creatinine, blood glucose, ECG, a clinical assessment of hydration and the Richmond Agitation Sedation Scale [29] were scheduled for daily assessments for safety reasons. Orthostatic BP tests were planned at day 5, 6 or 7 at 11.00 (approximately 3 h after drug intake), but occasionally done on day 4 if study drug was planned halted following protocol. All adverse events were recorded.

**Table 2:** Dosage plan for clonidine

Time	Safety	Dosage
Day 1 Loading doses	Systolic BP^a^ must be > 120 mmHg at inclusion (i.e. before the first loading dose). For any of the subsequent loading doses: If systolic BP is < 100 mmHg, HR^b^ < 50 beats/min, or if RASS^c^ is − 3 or less no more study medication will be given until the planned maintenance dose the next morning. If RASS is − 2, the treating physician has to assess if study medication will be given or not	75 μg every 3rd hour until maximum 4 doses, (e.g.: at 2, 5, 8 and 11 pm)
Day 2–7 Maintenance doses	If systolic BP is < 100 mmHg, HR < 50 beats/min, or if RASS is − 3 or less just before a planned dose, no study medication will be given until the next planned dose 12 h later. If RASS is − 2, the treating physician has to assess if study medication will be given or not	75 μg BID^d^, at 8–9 am and 8–9 pm

^a^BP = blood pressure

^b^HR = heart rate

^c^RASS = Richmond Agitation Sedation Scale

^d^BID = *bis in die* (i.e. twice a day)

### Measurements and procedures

Venous puncture for collection of plasma was scheduled 3 h after each drug intake on day 1, just before drug intake (between 8 am and 9 am on day 2 and day 5, 6 or 7) and 3 h after intake (C_max_) at day (4), 5, 6 or 7. Heparin tubes (4 ml) with blood were collected by venous puncture and centrifuged (2000G) for 10 min and two aliquots of at least 250 μl were stored in polypropylene tubes at − 80 °C pending analyses. Clonidine in plasma was determined by the method of Muller et al. [30] with modifications as described by Sulheim et al. [31]. The Data Monitoring Committee (represented by Leiv Otto Watne) was un-blinded to the randomisation, and identified the samples from the 10 patients that had received clonidine for plasma analyses.

### Statistical methods

Statistical analyses were performed in SPSS Statistics version 21 (IBM, Armonk NY) and Prism v7 (Graph Pad Software Inc., La Jolla, CA, USA).

Due to non-normally distributed data, non-parametric tests were used to compare continuous variables between the groups (Mann-Whitney tests) and to compare paired samples (Wilcoxon signed rank tests). To compare change from baseline to given time points between the groups, delta values were calculated and compared with Mann-Whitney U tests. A *p*-value of < 0.05 was considered statistically significant.

## Results

Between April 2014 and February 2017, of 407 inpatients considered to probably have delirium, 20 patients fulfilled the selection criteria and were included in LUCID and randomised to either clonidine (*n* = 10) or placebo (*n* = 10). Median age was 86 years (range 66–95), and 13 (65%) were women. Polypharmacy and use of other antihypertensive drugs were common (see Table 3), but none of the patients were using any known strong CYP2D6 inhibitors (i.e. fluoxetine/paroxetine [32], bupropion [33], quinidine [34], cinacalcet [35] or ritonavir [36]). See Table 3 for baseline characteristics.

**Table 3:** Baseline characteristics of study participants, *n* = 20

Variable	Clonidine, *n* = 10	Placebo, *n* = 10
Age, years, median (range)	85 (73–94)	88 (66–95)
Female, n/N (%)	6/10 (60)	7/10 (70)
Body mass index, kg/m^2^, median (range)	23 (19–29)	24 (17–28)
Creatinine at baseline, median (range)	78 (34–128)	88 (32–140)
Number of patients using other antihypertensive drugs^a^, n/N (%)	8/10 (80)	7/10 (70)

^a^Antihypertensive drugs used in the clonidine group (patients using the drug, n); metoprolol (5), furosemide (2), nifedipine (2), amlodipine (1), atenolol (1), bisoprolol (1), candesartan/hydrochlorthiazide (1), nitroglycerin (1) and tamsulosin (1)

### Plasma concentrations

The plasma concentrations in relation to time of drug administration are shown in Table 4. Three hours after the first dose of 75 μg clonidine, plasma concentration rose to median 0.35 μg/L (range 0.24–0.46). Median trough concentration before drug administration at day 2 was 0.70 (0.47–0.96) μg/L. After 4–6 days of clonidine treatment, median trough concentration (C_0_) was 0.47 (0.36–0.76) μg/L, rising to a level of 0.74 (0.56–0.95) μg/L (C_max_) 3 h after administration of the regular dose of 75 μg clonidine.

**Table 4:** Clonidine concentrations (μg/L) measured at day 1, day 2 and at steady state

Patient	Day 1				Day 2	Steady state Day 4,5 or 6 ^a^	
	Dose 1 C_max_	Dose 2 C_max_	Dose 3 C_max_	Dose 4 C_max_	C_0_	C_0_	C_max_
1	0.32	0.74	0.76		0.65	0.76	0.95
2	0.24	0.43			0.67	^b^	^b^
3				^c^	0.63		0.56
4	0.34	0.59	0.74	0.91	0.71	0.36	
5	0.39				0.77		0.61
6	0.27	0.41		^c^	0.47	0.41	
7	0.46	0.45	0.63		0.74	0.47	0.71
8		0.71		^c^	0.69		0.92
9	0.40	0.41	0.52		0.82	0.67	^b^
10	0.35	0.85	1.00		0.96		0.77
Median, μg/L	0.35	0.52	0.74	0.91	0.70	0.47	0.74
Range, μg/L	0.24–0.46	0.41–0.85	0.52–1.00		0.47–0.96	0.36–0.76	0.56–0.95
Missing, n	2	2	5	9	0	5	4

All samples were taken 3 h after administration of clonidine, except for C_0_ at day 2 and at steady state, taken just before administration of clonidine

^a^The samples at steady state were taken at day 4 (patients 5 and 10), day 5 (patients 3, 8 and 9), and day 6 (patients 1, 4, 6 and 7)

^b^The treatment was halted early (according to the protocol) and complete steady state measurements were not available

^c^4th dose not given (according to the protocol as patients were asleep)

### Hemodynamic changes during treatment

**Fig. 1:**
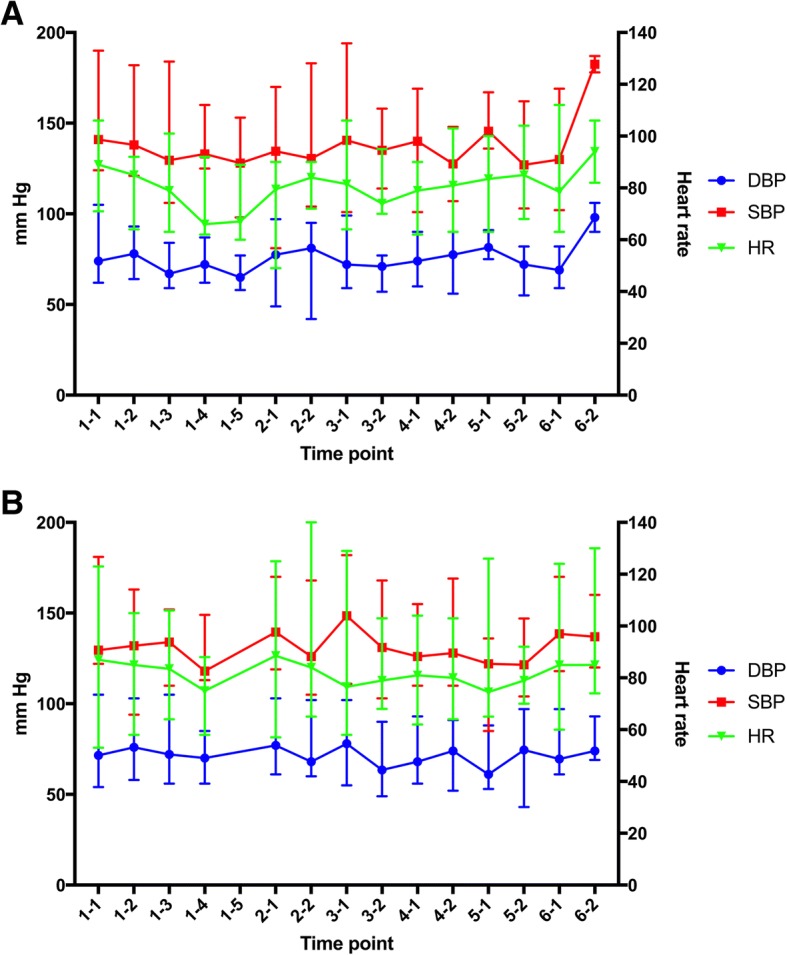
Panel **a** shows hemodynamic variables in the clonidine group and Panel **b** shows variables in the placebo group. Time points: First digit= day number, second digit = measurement at that day. At day two 2–1 is before morning dose and 2–2 is before evening dose. For panel **b**, time point 1–5 is not included in the figure as there were only measurements available from one patient. Median (range) values are displayed. DBP= Diastolic Blood Pressure SBP= Systolic Blood Pressure HR= Heart rate

Hemodynamic variables before and during clonidine treatment are presented in Table 5 and Fig. 1a and b for the clonidine and placebo groups, respectively. There was extensive individual BP variation in both treatment groups. The change in SBP from baseline to day 2 (delta values) was median − 16 (range − 61–40) in the clonidine group and median 7 (range − 40–47) in the placebo groups, but this difference was not statistically significant (median difference 21 mmHg (95%CI -12–44), *p* = 0.17).

**Table 5:** Hemodynamic variables before and during clonidine treatment

		Baseline	Day 1, 3 h after dose 1	Day 1, 3 h after dose 2	Day 1, 3 h after dose 3	Day 1, 3 h after dose 4	Day 2, before morning dose	Last measurement during treatment	Delta 1^a^	Delta 2^b^
Clonidine										
SBP^c^, mmHg	Median	141	138	**130**	133	**128** ^d^	135	137	−16	−10
	Range	124–190	121–182	106–184	125–160	98 – 153^d^	81–170	102 – 238^e^	− 61 - 40	−46 - 98
DBP^f^, mmHg	Median	74	78	**67**	72	65^d^	78	75	−5	−9
	Range	62–105	64–93	59–84	62–87	58–77^d^	49–97	56–109	−24 - 28	− 23 - 40
HR^g^, bpm^h^	Median	89	85	79	**66**	67^d^	84	76	− 6	−12
	Range	71–106	64–92	63–101	62–92	60–89^4d^	72–90	62–146	−41 - 18	− 32 - 61
Placebo										
SBP, mmHg	Median	130	132	134	118	170^i^	140	136	7	8
	Range	122–181	94–163	110–152	113–149	–	119–170	110–149	− 40 - 47	−56 - 19
DBP, mmHg	Median	72	76	72	70	85^i^	77	62	5	1
	Range	54–105	58–103	56–105	56–85	–	61–103	58–96	−27 - 19	−31 - 22
HR, bpm	Median	87	85	84	75	75^i^	84	80	1	1
	Range	53–123	58–105	64–106	58–88	–	65–140	64–130	−34 - 30	−36 - 36

Bold characters mark time points with a significant change in value from baseline (for SBP at 3 h after dose 2 and 4 (respectively *p* = 0.047 and *p* = 0.043), for DBP 3 h after dose 2 (*p* = 0.047) and for HR 3 h after dose 3 (*p* = 0.028)). The within group differences were otherwise not statistically significant

^a^baseline to day 2, before morning dose

^b^from baseline to last measurement during treatment

^c^SBP = systolic blood pressure

^d^Measurements from 5 of 7 patients that received the 4th loading dose

^e^One patient had SBP 238 mmHg at the last measurement during treatment, as a part of a hypertensive pulmonary oedema

^f^DBP = diastolic blood pressure

^g^HR = heart rate

^h^bpm = beats per minute

^i^Measurements only from one patient

Time points with a significant change from baseline were only found during the loading dosage regimen at day 1 within the clonidine group. A reduction in SBP from median 141 (range 124–190) mmHg at baseline to 135 (81–170) mmHg at day 2 in the intervention group was also noted, while no reduction was observed in the placebo group (Table 5). No adverse events were reported related to changes in BP and HR.

A test for orthostatic hypotension was performed in 11/20 patients. In the clonidine group, 1 of 6 patients had a fall in SBP > 20 mmHg, versus 1 of 5 patients in the placebo group. During the whole treatment period, a SBP < 100 mmHg was measured 3 times in the clonidine group (after the 4th loading dose and in the morning day 2 in patient no. 10, and in the morning day 2 in patient no. 5). SBP was also measured < 100 mmHg once in the placebo group. One patient (patient no. 10, morning day 2) had one measurement of HR 49, all other HR measurements were > 50 at all times and no patients had any relevant ECG changes.

**Fig. 2:**
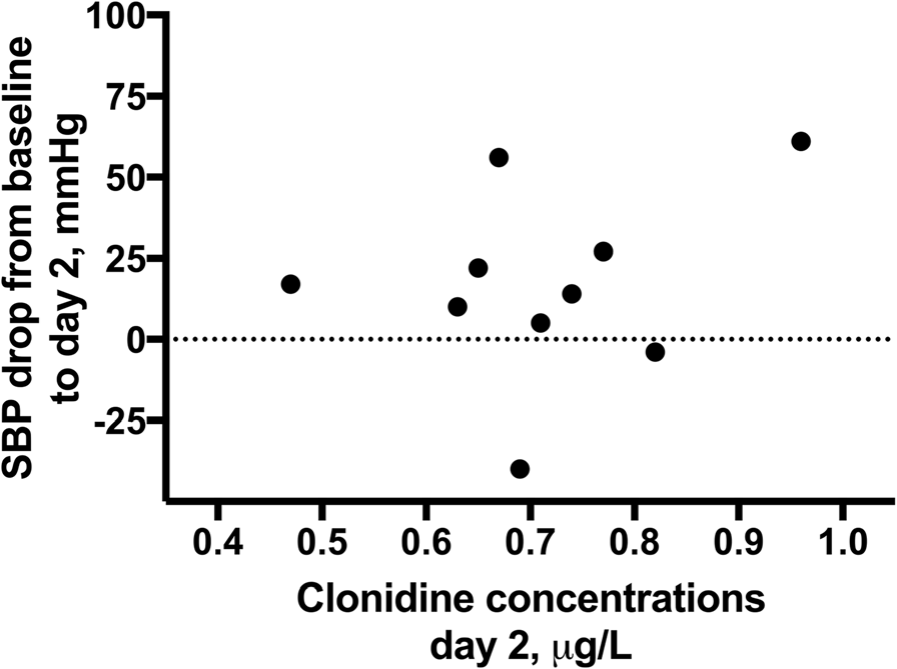
Scatterplot of clonidine concentrations and drop in SBP from baseline to day 2. There was no significant correlation between the clonidine concentrations and the level of drop of SBP from baseline to the morning at day 2. Pearson coefficient 0.271, *p* = 0.448

Patient no. 10 had a large drop in SBP from baseline to day 2 (from 142 to 81 mmHg). This patient also had the highest plasma concentration level after the third loading dose (1.0 μg/L), and before the drug administration on day 2 (0.96 μg/L), but kidney function and body mass index were within the normal range. However, as per the protocol, the patient did not receive the next dose, and the SBP and HR was within the accepted range during the rest of the treatment (4 days). Considering all 10 intervention patients, there was no correlation between the clonidine concentrations and the level of drop of SBP from baseline to the morning at day 2 (Pearson coefficient 0.271, *p* = 0.448 (Fig. 2)).

### Other adverse events

On the 5th day of treatment one patient in the clonidine group developed hypertensive pulmonary oedema (SBP 238 mmHg). According to the study protocol the study drug was halted. The Principal Investigator chose to report this to The Norwegian Medicines Agency as a Serious Unexpected Serious Adverse Reaction as causality may have been possible, although considering the known pharmacological effects of clonidine, hypertension caused by the treatment would not be expected. The patient died 2 weeks later and a follow up report was filed. In the placebo group two patients died during the hospital stay or shortly after discharge.

Regarding minor adverse events, two patients in both the clonidine and the placebo group reported dry mouth. One patient in the clonidine group experienced a fall during the treatment, but it was not considered related to hypotension and there was no orthostatic hypotension measured in this patient. There were no significant alterations in blood-glucose or significant episodes of sedation (RASS -3 or less) in either treatment group.

### Missing samples

27 of 70 (39%) scheduled blood samples were missing. For the planned sample after loading dose 4, 9 of 10 samples were missing (including 3 cases in which the patients never received the 4th dose). The other reasons for missing samples were: not all patients were willing to give repeated blood samples; some patients had veins that were difficult to puncture, making repeated punctures too uncomfortable; if the patients were asleep at midnight, we did not wake them up because it could worsen the delirium. Additionally, one patient (patient no. 2) was treated only for 3 days and gave no sample in steady state. No blood samples were lost after collection.

Of the 7 patients that received the 4th loading dose of clonidine, a control BP 3 h after this last dose was missing in 2 patients as they were asleep and waking them up might worsen the delirium.

## Discussion

The main findings of this study is that the dosage regimen described in the LUCID protocol, with a loading dose of 75 μg clonidine every 3rd hour up to a maximum of 4 doses on day 1 and further 75 μg twice daily, is generally adequate for target concentration of 0.3–0.7 μg/L. However, as the trough concentrations on day 2 were slightly high, a loading dose seems unnecessary and we propose that a revised dosage regimen of 75 μg twice daily from day 1 is both adequate and easier to administer. There was a trend that clonidine had an effect on BP and HR also in these geriatric medical patients, and these effects might relate to concentration levels. Our safety protocol with measurements of BP and HR before administering clonidine to this patient group thus proved both necessary and adequate.

The step-wise regimen with repeated doses of 75 μg every 3rd hour, up to a maximum of 300 μg the first day, was effective and resulted in plasma concentration levels at the higher end of the expected therapeutic range. We aimed for plasma concentration levels between 0.3 and 0.7 μg/L, and calculated theoretically that C_max_ at day one should be lower than 1.2 μg/L [22]. No measurements showed higher values than 1.0 μg/L, but many samples are missing after the last dose at day 1, and so we cannot rule out that some patients had concentrations above 1.2 μg/L. Also, the trough concentration at day 2 was at the higher end of our pre-specified target concentration, and would rise further after the next dose (even though this was not measured in this study). The three patients that only received 3 loading doses had among the lower values at day 2 as expected. A few patients had some measurements higher than 0.7 μg/L, and no patients had lower levels than 0.3 μg/L on day 2. Based on our findings of some concentration levels higher than expected and trough concentrations in the higher end at day 2, we suggest a loading dose is unnecessary and a dosage regimen of 75 μg twice daily from day 1 is both adequate and easier to administer.

Examining the response from the very first dose of 75 μg clonidine, the median plasma concentration level (0.35 μg/L) was already within our pre-specified expected range. This finding is in line with previously published studies of plasma concentrations of clonidine in younger adults [23] where it was shown that a single dose of 75 μg clonidine gave concentration max of 0.285 (+/− 0.001) ng/ml. That study seemingly had a lower inter-patient variability than we found, possibly due to a healthier, younger population. We chose a single time-point for C_max_ concentration measures (3 h post dose). As the time (T_max_) from intake to C_max_ varies inter-individually, our variability might simply be a reflection of this. Several repeated samples (e.g. from in-dwelling cannulas) could have given more accurate measures of T_max_ and thus C_max_ for every patient. However, in our population of frail, elderly patients even more blood samples, or the use of in-dwelling cannulas, would raise both practical and ethical issues. Also, we assume that by collecting samples at a time corresponding with a late T_max_ (i.e. 3 h) we will still be relatively close to C_max_ even in patients who may have a shorter T_max_, given the relatively long half-life of clonidine (between 5 to 25.5 h).

The clonidine concentration levels measured at steady state (day 4–6) were also within the target range. After the initial loading doses, clonidine 75 μg twice daily was sufficient to reach trough concentrations (C_0_) at median 0.47 μg/L, rising to a median level of 0.74 μg/L (C_max_) after intake of another 75 μg clonidine. These results are also in line with previous studies. In a study of adolescents with chronic fatigue syndrome, a dosage of 50 μg twice per day for 14 days resulted in median C_0_ at 0.21 μg/L, rising to median 0.41 μg/L (C_max_) 2 h after administration of one regular dose of 50 μg [26]. In subjects receiving oral clonidine 100 μg twice per day for 6 weeks, plasma concentration ranged between 0.4 and 0.7 μg/L (2 h after intake of 100 μg) [25]. Another study found that a single dose of 75 μg gave a C_max_ of 0.66 μg/L after achieving steady state with two 75 μg doses [37].

Clonidine has well known antihypertensive effects and it also lowers the heart rate [37]. The maximum hypotensive effect and degree of bradycardia are related to dose and peak plasma concentrations [23]. Therefore, the hemodynamic changes with a trend of lower BP and HR during clonidine treatment were as expected as we reached the assumed therapeutic concentrations. The changes in BP and HR in patients treated with clonidine were not significantly larger than in the placebo group. Wide 95% confidence intervals indicate that this is likely due to the low number of participants as the trend was clearly present in the clonidine group and not in the placebo group. Our main goal was, however, not to formally establish differences in BP between the clonidine and the placebo group. In order to ensure sufficient statistical power for this comparison, a much larger study would have been necessary. It has previously been shown that there is a correlation between plasma concentration levels and drop in SBP [25]. In this group as a whole we could not statistically confirm this, but again this might be due to the small sample size.

Also, a great variability in blood pressure and heart rate values would be expected in a geriatric hospital population due to the natural course of the illnesses and other treatment received. Notably, even if the median blood pressure and heart rate values were lower in the patients that received clonidine, the values were not below that what would be generally considered safe and adverse symptoms were not reported related to changes in BP and HR. So, even if the plasma concentration levels of clonidine occasionally were higher than 0.7 μg/L, this dosage regimen did not have any hemodynamic effects considered unsafe.

Large variability in the half-life of clonidine has previously been reported, and for elderly patients who tend to have an increase in volume of distribution of lipid soluble drugs [38] (like clonidine) such an increase of half-life would be expected to be more frequent. Half-life was however not estimated in this study. Effects of polypharmacy and interactions related to metabolizing enzymes are another source of variability and heterogeneity. To avoid known interactions, a few drugs were listed as exclusion criteria and to our knowledge; none of the medications used in our patients are strong CYP2D6 inhibitors. However, several other antihypertensive medications were in use affecting the BP variation. Also, in this elderly population a varying degree of reduction of renal and hepatic clearance must be expected. Dose adjustments based on renal function is recommended, and thus an eligibility criterion of eGFR > 30 was chosen as it was not feasible to include individual dose adjustments in our protocol. For hepatic function, the manufacturer suggests no dose adjustments. Accordingly, no dose adjustments or safety measures of liver function were included.

Patient no. 10 illustrates the difficulties in predicting individual effects on BP. The patient had a large reduction in SBP during the first day of treatment, but both the kidney function and the body mass index were within the normal range, and the patient did not otherwise differ from the other patients receiving clonidine. Many of these issues are not unique to clonidine, but illustrate some of the challenges in administering drugs in the geriatric population. This emphasizes the importance of clinical efficacy measurements for safety assessments.

The reasons for missing samples illustrate some of the feasibility issues with intensive follow-up studies in this population. Despite signalising a positive attitude at inclusion, not all patients were willing to give repeated blood samples. In other cases, we had to consider what was best for the patients (i.e. veins that were difficult to puncture, making repeated punctures too uncomfortable; and the risk of worsening delirium if the patient was asleep). Pharmacological studies are rarely performed in elderly patients, and in future studies these challenges must be taken into account in the study design both regarding sample schedules (e.g. timing of samples; which are the most crucial and which can be ‘optional’) and sample size (anticipating the proportion of samples likely to be missed).

This study is part of a well-designed RCT with a pre-published protocol. The study included a real-life, placebo-treated control group in the assessment of hemodynamic changes. There was a clear pre-defined prediction of plasma concentration levels, based on theoretical calculations. The patients were monitored very closely; safety and optimal clinical care of the patients was the priority. Some limitations of the study need to be acknowledged. Some planned plasma concentration samples are missing. The sample size was small. Additionally, because of the strict exclusion criteria, the external validity of our findings might be limited, at least regarding the pharmacodynamic effects of clonidine.

## Conclusion

The main finding of this study is that a dosage regimen, as described in the LUCID protocol, with a loading dose of 75 μg every 3rd hour up to a maximum of 4 doses on day 1 and further 75 μg twice daily, seems safe, with the limitation that this is based on a small sample size. For a target concentration of 0.3–0.7 μg/L, a loading dose is unnecessary and we propose that a dosage regimen of 75 μg twice daily from day 1 is both adequate and easier to administer. There was a clear trend that clonidine has an effect on BP and HR, which might relate to concentration levels, and we believe a safety protocol with measurements of BP and HR before administering clonidine to this patient group is both a necessary and a sufficient precaution. We also found that this geriatric population does have a higher inter-patient variability of plasma concentration levels compared to previous studies done in healthier, younger populations, which illustrates the difficulties of geriatric pharmacological treatment and supports the need for clinical efficacy measurements for these patients.

## Abbreviations

BP: Blood pressure
bpm: Beats per minute
C_0_: Trough concentration
C_max_: Maximum plasma concentration
DBP: Diastolic blood pressure
HR: Heart rate
ICU: Intensive care unit
LUCID: The Oslo Study of Clonidine in Elderly Patients with Delirium
MAP: Mean arterial pressure
RCT: Randomised controlled trial
SBP: Systolic blood pressure
T_max_: Time from intake to C_max_
